# Language use and suicide: An online cross-sectional survey

**DOI:** 10.1371/journal.pone.0217473

**Published:** 2019-06-13

**Authors:** Prianka Padmanathan, Lucy Biddle, Katherine Hall, Elizabeth Scowcroft, Emma Nielsen, Duleeka Knipe

**Affiliations:** 1 Population Health Sciences, Bristol Medical School, University of Bristol, Bristol, United Kingdom; 2 Avon & Wiltshire Mental Health Partnership NHS Trust, Bristol, United Kingdom; 3 Samaritans, Surrey, United Kingdom; 4 Self-Harm Research Group, School of Psychology, University of Nottingham, Nottingham, United Kingdom; Middlesex University, UNITED KINGDOM

## Abstract

**Background:**

There has been a recent focus on language use in relation to suicide, with concerns raised about the potential to cause distress, perpetuate stigma and discourage help-seeking. While some terms are promoted as more sensitive than others, empirical research exploring the views of people affected by suicide to inform academic and media guidelines is lacking.

**Methods:**

An anonymous, cross-sectional online survey was promoted opportunistically via online channels. Participation was requested from adults affected by suicide. Participants were asked to rate descriptors pertaining to suicidal behaviour according to perceived acceptability. A descriptive analysis of quantitative data was conducted alongside thematic content analysis of free-text data.

**Outcomes:**

There were 2,719 responses, of which 1,679 (61·8%) were complete. Of phrases describing non-fatal suicidal behaviour, “attempted suicide” had the highest median acceptability score. Of phrases describing fatal suicidal behaviour, “took their own life” and “died by suicide” had the highest median acceptability scores. The scores for “commit suicide” were most variable and spanned the range of acceptability scores. Free text data illustrated the nuances in decision-making.

**Interpretation:**

Variation in opinion exists amongst people affected by suicide regarding most phrases, often depending on contextual factors. “Attempted suicide”, “took their own life”, “died by suicide” and “ended their life” were however considered most acceptable. We argue that academic and media guidelines should promote use of these phrases.

## Introduction

The importance of language in reflecting and shaping assumptions has long been recognised.[1] Research has demonstrated the influence of language on thoughts and memory.[2,3] Furthermore, substitution of single words alone have been found to have an effect on attitudes and behaviours.[4,5] With regards to the field of mental health, in which stigma may discourage help-seeking and impact upon experiences of care, close attention is often given to language used. [6,7]

Suicide is highly stigmatised. Amongst people who have lost a significant other due to suicide, stigma has been associated with increased social withdrawal and reduced psychological and somatic functioning.[8] People bereaved by suicide have highlighted that the word “commit” is most commonly used in conjunction with a criminal act, resulting in a negative connotation of immorality, which is now inconsistent with the legal status of suicide in most countries globally.[9–12] Consequently, use of the phrase “commit suicide” in the media and in academia has been discouraged.[13–15] Most recently a letter, signed by over 140 public figures and widely publicised in the British media, advised against use of this phrase in the context of responsible media reporting.[16–18] Alternative phrases such as “died by suicide” and “took one’s own life” have been suggested. However, these phrases each have their own implications; for example “died by suicide” may remove a person’s agency in suicide and “took one’s own life” omits the word “suicide” itself.[19,20] Careful consideration is therefore required when making decisions about language use, but to date there has been little research to guide this.

This study explores the views of people affected by suicide with regard to language used in relation to suicidal behaviour. People were considered to have been affected by suicide if they or someone they knew had experienced thoughts of suicide or attempted suicide, or if anyone they knew had died by suicide.

## Methods

To avoid confusion, the phrases “non-fatal suicidal behaviour” and “fatal suicidal behaviour” have been used to group the terms evaluated in this paper. These phrases were not evaluated in the study due to their infrequent use. We therefore acknowledge that use of these phrases may succumb to some of the very problems being discussed.

### Study design and participants

A pragmatic survey was developed and piloted by the authors, some of whom have been affected by suicide or self-harm, to explore language use in relation to suicidal behaviour (S1 File). A participant information page was included at the beginning of the survey and consent was obtained. The survey included 33 questions. Standard baseline data were collected on the following: participants’ age group, gender, level of education and country of residence. The way in which participants had been affected by suicide was also included to enable exploration of its effect on results. A five-point Likert-type scale was chosen to measure and compare attitudes to different descriptors of suicidal behaviour. Descriptors were included if they had been encountered by any of the co-authors within the academic, clinical and charity settings in which they work. Following feedback from the reviewing ethics committee, descriptors were organised into separate questions based on their relevance to whether or not a person had died. Participants were therefore asked to rate phrases to describe a situation “where a person has performed an act of suicide or self-harm but has not died”, giving a score between 1 (“not acceptable”) and 5 (“acceptable”). They were then asked to rate phrases to describe a situation “where a person has performed an act of suicide or self-harm and has died”. To augment findings, drop-down lists of descriptors were included to ascertain which terms participants believed to be most and least appropriate. Free-text questions were included to understand participants’ reasons for their choices and to allow alternative descriptors to be suggested. Ethical approval was granted by the University of Bristol Faculty of Health Sciences Research Ethics Committee.

The anonymous survey was hosted online (SurveyMonkey) between 1^st^-31^st^ August 2018. The survey was promoted through the authors’ own national and international research networks as well as lived experience and charitable organisations in the United Kingdom (UK), Australia and United States of America (USA), using email, Twitter, Instagram, Reddit, Facebook and organisation websites. Recruitment messages specified that participants should be over 18 years of age and have been affected by suicide. No other restrictions were applied.

### Statistical analysis

A primary descriptive analysis of quantitative data was conducted on all complete responses of those personally affected by suicide and living in majority native English-speaking countries [21]. Data were analysed using Stata (version 15.1). Participants were considered to have been personally affected by suicide if they had experienced thoughts of suicide, attempted suicide, or if anyone they knew personally had experienced thoughts of suicide or attempted or died by suicide. Participants whose only experience of suicide was through their work, for example working in a professional or voluntary capacity with someone who had attempted or died by suicide, were excluded in the primary analysis (*n* = 33). A sensitivity analysis was conducted on all (including incomplete) responses of the same population (S2 File). Due to the skewed nature of the results, medians and interquartile ranges were calculated and the data were summarised using box plots. As acceptability scores were highly varied for the phrase “commit suicide”, the quantitative responses of those who selected “commit suicide” as the term they considered most appropriate or least appropriate option were explored.

A secondary analysis was conducted comparing the acceptability scores for terms to describe fatal suicidal behaviour between people who: (1) experienced thoughts of suicide or attempted suicide themselves, (2) personally knew someone else who had experienced thoughts of suicide or attempted or died by suicide, and (3) worked or volunteered with people affected by suicide. Participants were excluded from this analysis if they fit into more than one group. A supplementary analysis (S3 File) explored the results of participants living in the UK, Australia and the USA separately.

### Free-text analysis

Thematic content analysis was performed on free-text data from complete responses regarding the terms emerging as most and least appropriate in the quantitative analysis. For example, as “attempted suicide” received the highest acceptability scores, the free-text responses of those who selected this term as most appropriate were analysed. “Topped themselves” had the lowest median and interquartile range scores of descriptors of fatal suicidal behaviour. However, due to infrequent use it was considered more useful to analyse the free text data from the term with the second lowest acceptability scores, “successful suicide”. Free text analysis was also performed on the complete responses of participants from English-speaking countries who selected “commit suicide” as the term they considered most or least appropriate to use, to understand the highly variable scores for this term and because this has been the focus of much of the debate around language.

For each term, two authors (P.P or K.H. in conjunction with L.B.) carried out an independent open coding of the first 50 responses to identify themes. Codes were compared and any differences discussed until consensus was reached and a coding frame derived. A single author (P.P. or K.H.) then used the frame to code all remaining responses for each term. To provide a broader context, complete coding of all terms was also performed for the first free-text question (preferred term for non-fatal suicidal behaviour).

### Role of the funding source

Funding from a National Institute of Health Research Academic Clinical Fellowship was used to host the survey online. The Wellcome Trust Institutional Strategic Support Fund enabled Open Access publication. The work was also supported by the Elizabeth Blackwell Institute for Health Research, the Economic and Social Research Council [ES/J500100/1] and MRC Addiction Research Clinical Training programme. The funders had no role in the design of the study and collection, analysis, and interpretation of data and in writing the manuscript. All authors had full access to all the data in the study and had final responsibility for the decision to submit for publication.

## Results

There were 2,719 responses, of which 1,679 (61·8%) were complete. Of the complete responses, 1,437 participants (95·1%) lived in English-speaking countries: 815 in the UK, 339 in Australia and 208 in the USA. Knowing a close friend or relative who had attempted or died by suicide was the most common way in which participants had been affected by suicide (*n* = 879; 52·4%). Table 1 outlines the baseline characteristics of respondents. People with incomplete quantitative responses were more likely to be younger, male, living in non-English speaking countries and more likely to have a lower level of education compared to those with complete quantitative responses.

**Table 1:** Baseline characteristics.

	Categories	No. of complete quantitative responses (%)	No. incomplete quantitative responses (%)
**Age**	18–29 years old	571 (37·8)	632 (52·5)
	30–49 years old	588 (38·9)	407 (33·8)
	50–64 years old	306 (20·3)	140 (11·6)
	≥65 years old	46 (3·0)	24 (2·0)
**Gender**	Female	1151 (76·2)	820 (68·3)
	Male	292 (19·3)	328 (27·3)
	Transgender female	11 (0·7)	9 (0·8)
	Transgender male	12 (0·8)	10 (0·8)
	Gender non-conforming	25 (1·7)	18 (1·5)
	Other	13 (0·9)	8 (0·7)
	Prefer not to say	7 (0·5)	8 (0·7)
**Highest level of education**	Primary Education	28 (1·9)	48 (4·0)
	Secondary/Further Education	475 (31·4)	444 (37·0)
	Higher Education	974 (64·5)	685 (57·1)
	Other	34 (2·3)	23 (1·9)
**Country of residence**	English-speaking countries	1437 (95·1)	1056 (89·6)
	Non-English-speaking countries	74 (4·9)	123 (10·4)
Affected by suicide*	A close friend or relative attempted or died by suicide	879	599
	An acquaintance attempted or died by suicide	427	314
	I have worked in a professional capacity (e.g. as a practitioner) with someone who has attempted or died by suicide	281	167
	I have attempted suicide	485	313
	I have experienced thoughts of suicide, but not acted on them	720	610
	None—I have not had any experience of suicide	0	39
	Other	91	142

Using chi2 test, p<0·0001 for age, gender, education level and country of residence

*more than one option could be selected, category for no experience of suicide not included in chi2 test

### Non-fatal suicidal behaviour

**Fig 1:**
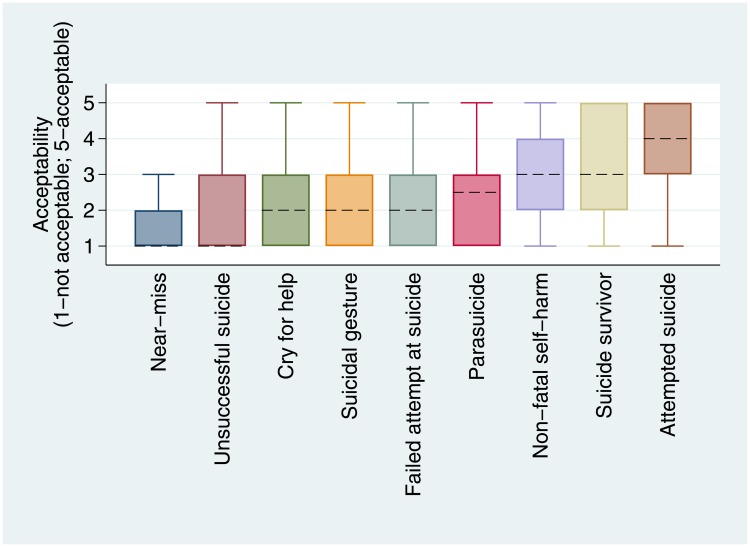
Box plots of acceptability scores for descriptors of non-fatal suicidal behaviour (excludes outliers; ordered by medians, denoted by dashed line; 1 = unacceptable 5 = acceptable).

“Attempted suicide” had the highest median score (4; IQR = 3–5) and “near miss” had the lowest median and interquartile range scores (1; IQR = 1–2) out of descriptors of non-fatal suicidal behaviour (Fig 1). The same terms had the highest and lowest acceptability scores in the sensitivity analysis including incomplete data (S2 File). The interquartile range of both “non-fatal self-harm” and “suicide survivor” spanned both acceptable and unacceptable scores. All other descriptors were deemed ‘unacceptable’ by most participants.

Of the 845 participants who selected “attempted suicide” as the most appropriate term for non-fatal suicidal behaviour, 697 provided a free-text comment. Participants most commonly cited the following reasons: perceived accuracy, clarity and neutrality (Panel 1). When participants referred to the content and meaning of the phrase, they most commonly stated that it was non-euphemistic and reflected the motive and the seriousness of the behaviour, without imposing an explanation. Opinion was divided regarding the tone of the phrase, with some stating that they selected the phrase as it sounded clinical, whilst others preferring that it was not too clinical.

“It is an accurate description without the subjective overtones of some of the others. It does not attempt to interpret the reasons behind the person's action which are known only to him/her.”

(≥ 65years, close friend or relative attempted or died by suicide, has attempted suicide themselves)

Of 287 people who selected “near miss” as the least appropriate term for non-fatal suicidal behaviour, 233 provided a free-text comment. Comments focussed more on the meanings associated with the phrase than the general characteristics, although some did highlight the vagueness or ambiguity of the phrase. The most common reason for considering the phrase least appropriate was that it understated the seriousness of the event. Participants also suggested that it did not reflect suicidal intent and was insensitive and disrespectful. Implied failure or negativity in relation to surviving was also noted, which a few participants suggested could incentivise further suicidal behaviour.

“Sounds like the fact the suicide didn’t succeed is a “miss” or bad thing. This is a horrible term to use.”

(18–29 years, close friend or relative attempted or died by suicide, an acquaintance had attempted or died by suicide, has experienced suicidal thoughts themselves but had not acted on them)

“It’s an expression which is dismissive of the persons suffering. Making their pain insignificant. Fobbing off the severity of their situation.”

(50–64 years, close friend or relative attempted or died by suicide, has experienced suicidal thoughts themselves but had not acted on them)

### Fatal suicidal behaviour

**Fig 2:**
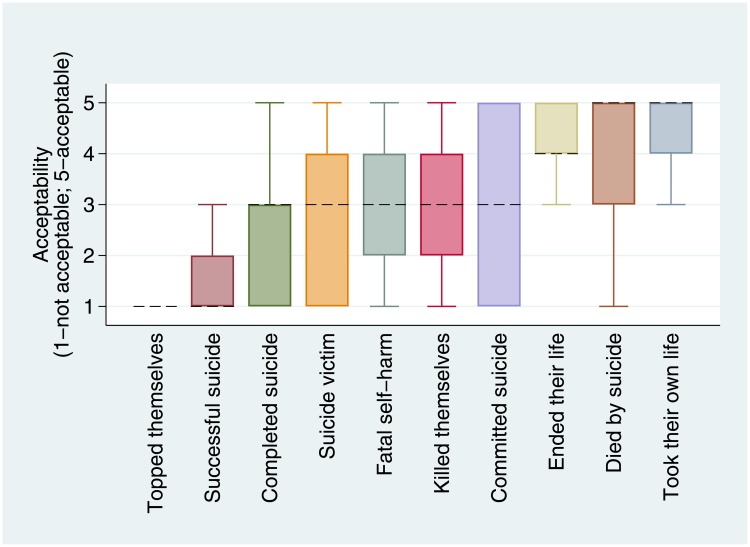
Box plots of acceptability scores for descriptors of fatal suicidal behaviour (excludes outliers; ordered by medians, denoted by dashed line; 1 = unacceptable 5 = acceptable).

Of descriptors of fatal suicidal behaviour, “took their own life” (5; IQR = 4–5) and “died by suicide” (5; IQR = 3–5) had the highest median scores, “topped themselves” (1; IQR = 1–1) and “successful suicide” (1; IQR = 1–2) had the lowest median scores (Fig 2). These findings were replicated in the sensitivity analysis (S2 File). The interquartile range of several phrases spanned both acceptable and unacceptable scores: “suicide victim”, “fatal self-harm”, “killed themselves”, and “committed suicide”. The scores for “committed suicide” were most varied, spanning the entire range.

There were 211 participants who selected “took their own life” as the most appropriate term for fatal suicidal behaviour, 169 of whom provided free-text comments. A reflection of an individual’s agency was most commonly cited as the reason for choosing the term. Participants also frequently stated that they had selected the phrase because it was descriptive and softer in tone. In contrast to opinion regarding “attempted suicide”, some participants specifically highlighted that they preferred that it did not include the word suicide.

“It addresses the fact that they chose to do it. Without it sounding too harsh. I use this term when talking about my friend. It keeps her human.”

(30–49 years, close friend or relative attempted or died by suicide, has worked in a professional capacity with someone who has attempted or died by suicide, has experienced suicidal thoughts themselves but had not acted on them)

“After a number of attempts over many years Mum took her own life. I feel it is important to recognise her agency without judgement even although she was experiencing a severe depressive episode at the time.”

(50–64 years, close friend or relative attempted or died by suicide, an acquaintance attempted or died by suicide, has worked in a professional capacity with someone who has attempted or died by suicide)

“I hate the word suicide because it freaks people out, so for me this is the softest version.”

(18–29 years, close friend or relative attempted or died by suicide, an acquaintance attempted or died by suicide, has worked in a professional capacity with someone who has attempted or died by suicide, has attempted suicide themselves)

“*The word suicide is still associated with illegality and negative morality*…”

(≥ 65years, close friend or relative attempted or died by suicide, an acquaintance attempted or died by suicide, has experienced suicidal thoughts themselves but had not acted on them)

Of the 381 participants who considered “successful suicide” the least appropriate term for fatal suicidal behaviour, 327 provided a free-text comment. The majority of these participants attributed this to its positive framing of suicide. Some specified that the term inappropriately celebrated suicide or portrayed it as an achievement. A few raised concerns that this could encourage suicide. The term was considered insensitive and distressing to those bereaved by suicide.

“What is successful about not getting help and support, and not seeing a reason to live????”

(50–64 years, close friend or relative attempted or died by suicide, an acquaintance attempted or died by suicide, has worked in a professional capacity with someone who has attempted or died by suicide, has experienced suicidal thoughts themselves but had not acted on them)

“I think this essentially glorifies the suicide, making it seem like something to aim for or have a goal. Suicide is a last resort for people, it’s not something to celebrate as a success”

(30–49 years, close friend or relative attempted or died by suicide, has attempted suicide themselves)

“"Successful" has positive connotations which I feel is completely inappropriate. There is absolutely no success and nothing positive for anyone affected by suicide—not for families and friends, nor for the victim themselves. It is a result of unbearable distress and desperation.”

(18–29 years, close friend or relative attempted or died by suicide, has experienced suicidal thoughts themselves but had not acted on them)

**Fig 3:**
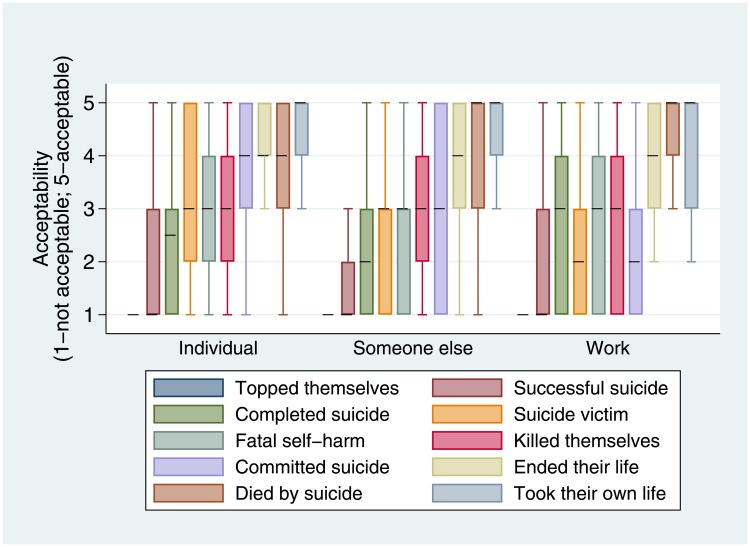
Box plots of acceptability scores for descriptors of fatal suicidal behaviour by manner in which affected by suicide (excludes outliers; medians denoted by dashed line; 1 = unacceptable 5 = acceptable).

Most participants were affected by suicide through more than one type of experience. Of complete responses of those living in majority native English-speaking countries, 298 (20.7%) participants had been affected by suicide exclusively through their own experiences (“individual”), 319 (22.2%) participants had been affected solely through those of someone they knew outside work (“someone else”) and 33 (2.3%) participants’ only experience was of working or volunteering with people affected by suicide (“work”). The descriptors considered most and least acceptable were the same for each group. However some variation in medians and interquartile ranges for a number of descriptors can be observed (Fig 3).

Opinion about the phrase “committed suicide” was most divided amongst people who had been affected by suicide through someone they knew (3; IQR = 1–5). Those who had been affected by suicide solely through their own experiences more commonly found it to be acceptable (4; IQR = 3–5) compared with those whose experience of suicide was exclusively through work or volunteering (2; IQR = 1–3).

Amongst participants who selected “commit suicide” (n = 367) as the most appropriate term, “took their own life” (5; IQR = 4–5), “ended their life” (4; IQR = 3–5), “died by suicide” (4; IQR = 3–5) and “killed themselves” (4; IQR = 3–5) were also generally considered acceptable alternatives (S4 File). Of the 274 participants who provided a free-text comment, most commonly cited reasons of accuracy and common usage for choosing “commit suicide” as most appropriate. Some disputed the idea that the term implies criminal undertones. “Commit suicide” was not selected as most appropriate by anyone who had experienced suicide through work or volunteering only. The free-text responses were similar between people who had been affected by suicide through their own experiences and through the experiences of others. More people who had been affected by suicide through their own experiences cited the clarity of the phrase “commit suicide” as their reason for choosing it. More people who had been affected by suicide through the experiences of someone they knew favoured it for being ‘to the point’ and non-euphemistic.

“because by the very definition of "committed", it is EXACTLY what my son did. It has NO implications of being something unlawful or an act of crime, as many suicide "experts" will try to tell you. Political correctness gone bat s*** crazy. Makes me SOOOOOOOOO angry. Do not tell ME what my son did! *Don’t you dare "correct" me!”*

(50–64 years, close friend or relative attempted or died by suicide)

“They were committed to the act of not living. I know committing is supposed to have a criminal content, but we don’t think of that when people commit to marriage, etc so it’s acceptable to me. I use it the term.”

(50–64 years, close friend or relative attempted or died by suicide)

“It’s the most common term to use, and I find it pretty neutral”

(18–29 years, has experienced suicidal thoughts themselves but had not acted on them)

“It states the situation as it is and it presents the realest explanation, it us also the least offensive because it is what the person did, it is also a formal way to say it which is better than the others on the list”

(18–29 years, has attempted suicide)

There were 167 free-text comments from the 181 participants who selected “commit suicide” as the least appropriate terminology. Respondents most commonly cited the criminal connotation associated with the phrase. A number also perceived it to be outdated. No difference was noted between those who had been affected by suicide only through their own experiences and those who had experienced suicide through others, personally or professionally.

*“My son was not a criminal*!*”*

(50–64 years, close friend or relative attempted or died by suicide)

“It is too shocking a phrase to hear and say. It is filled with emotional weight that stops conversation.”

(50–64 years, close friend or relative attempted or died by suicide)

“This term was created when suicide was a crime. Continuing to use it just perpetuates suicide is a crime and isn’t compassionate language.”

(18–29 years, has attempted suicide)

### Alternative phrases

Of the 1,679 complete quantitative responses, 188 participants provided suggestions of alternative phrases to describe suicidal behaviour, although many indicated that these were not necessarily preferred options. The most common suggestion was inclusion of a statement reflecting the intention of the person who has died. Most of those who advocated for this specifically suggested “chose to” or “decided to” before a descriptor. However, other participants expressed a preference to avoid reference to an individual’s agency because this implied free choice.

“…I wouldn’t say chose to end their life, because at that point it doesn’t seem like you have a choice.”

(*30–49 years*, close friend or relative attempted or died by suicide, has experienced suicidal thoughts themselves but had not acted on them)

“The word suicide suggests an act that someone has made the choice to carry out. So many people have negative views of this word (selfish, coward etc.). My Dad died by suicide and he was very unwell, his judgement and intent was impacted by his mental health. It wasn’t a choice for him. But people who hear suicide don’t see it that way.”

(*18–29 years*, close friend or relative attempted or died by suicide)

The second most common response related to the inclusion of an underlying cause of death within the phrase rather than the method. Suggestions included “died by brain disease”, “mental illness”, “hopelessness” or “family rejection”. One participant summarised reasons for and against this approach.

“It’s not a complete swap-out, but I do wonder why, for people with known mental illness preceding their death by suicide, we don’t say they died from mental illness. To me it’s like cancer. Technically someone might die from a complication of cancer, like pneumonia, but we say they died from cancer. If I die from suicide, I died from depression. However, I can see that that may be a very individual and complex thing, and suicide is often multifactorial.”

(18–29 years, has worked in a professional capacity with someone who has attempted or died by suicide, has attempted suicide themselves)

A number of participants described their concerns regarding the avoidance of using the word “suicide”.

“I think avoiding the word suicide all together is dangerous in itself. We’re isolating those that might be having those thoughts and telling them it’s not ok to reach out and ask for help and making them feel alone.”

(30–49 years, close friend or relative attempted or died by suicide)

“My brother committed suicide and my sister attempted suicide. I don’t think we should be scared of using the word suicide but maybe as a society we should be more open.”

(50–64 years, close friend or relative attempted or died by suicide)

Other ideas and phrases highlighted were: the use of language differs depending on the person and the context; “died” or “passed away” without the use of the word “suicide”; use of the word “battle” e.g. “lost the battle with depression”; “victim” e.g., “victim of suicide/ mental health”; “suicided”; “ended suffering”; using “died of” or “from”, rather than “by”, suicide.

### Summary of free-text data

Overall, participants’ responses to free text questions indicated a preference for terms that were perceived to be factual, clear, descriptive, commonly used and non-emotive. Non-judgment was frequently cited as an important characteristic, although participants also highlighted a range of judgments associated with terms considered appropriate: reflecting the seriousness of the behaviour, evoking compassion, refraining from blame whilst valuing life. Where participants’ responses considered the potential consequences of phrases, appropriate terms were those considered to be non-stigmatising, respectful and validating, while some thought that inappropriate terms could trigger suicidal behaviour (Table 2).

**Table 2:** Themes and codes commonly emerging from free text analysis.

	Most appropriate term	Least appropriate term
**General characteristics**		
Accuracy	Factual	
Comprehensibility	Clear	Ambiguous
Descriptiveness	Descriptive	Vague, over-simplistic
Familiarity	Common parlance	
**Tone and content**		
Clinical or personal	Clinical vs. Not too clinical	Casual, detached, dehumanising
Emotional content	Non-emotive	Highly emotive
Direct acknowledgement of “suicide”	Softer vs. To the point	Avoidant
**Meaning**		
Neutrality	No judgment	Judgment
Specific meanings	Reflects individual's decision Reflects seriousness Evokes compassion Does not imply criminality/blame Values life	Downplays agency Trivialises Lacks compassion Frames living or suicide as failure Frames suicide as success
**Potential consequences**		
Effect on perceptions of suicidal behaviour	Non-stigmatising	
Effect on person engaging in suicidal behaviour	Validates emotions	Encouraging suicidal behaviour
Effect on those affected by suicidal behaviour	Respectful	Disrespectful

## Discussion

### Main findings

This is the first study to investigate preferred language use in relation to suicidal behaviour of those affected by suicide. The majority of participants found “attempted suicide” acceptable to describe non-fatal suicidal behaviour, and “took their own life”, “died by suicide” and “ended their life” acceptable to describe fatal suicidal behaviour.

Opinion regarding the phrase “commit suicide” was highly variable. Those who had been affected by suicide solely through their own experiences more commonly found it acceptable compared with those whose experience of suicide was exclusively through work or volunteering. Opposing views were, however, present in both groups. Reasons for choosing “commit suicide” as a preferred phrase for suicidal behaviour were that it was accurate, widely used and not perceived to have criminal undertones. However, those who selected “commit suicide” as the least appropriate terminology most commonly cited criminal connotations as the reason for this decision.

The findings demonstrate variations in opinion, particularly in terms of contextual factors such as how the individual has been affected by suicide and perceived intent of the act and whether this emerged from free choice. The former is highlighted by the term “suicide survivor” scoring more highly amongst people who had been affected by suicide solely through their own experiences, compared to those affected through the experiences of others. Variation in reasoning was also noted amongst people who considered the same term most appropriate, for example some endorsing the phrase “attempted suicide” for being clinical and some preferring it for precisely the opposite reason. This demonstrates the importance of reflective communication in one-to-one discussions. Where this is not possible in the media and academia, the findings indicate a number of terms that are more consistently considered acceptable by most people affected by suicide, and the factors that individuals consider when appraising this terminology.

### Strengths and limitations

This is the first large-scale study with international representation, which actively recruited those affected by suicide to investigate their preferred language use in relation to suicidal behaviour. The results should however be considered in light of a number of limitations. First, the study aimed to investigate language use in relation to suicidal behaviour, but referred to both suicide and self-harm (fatal and non-fatal) due to the use of this terminology amongst academics and national guidelines.[22] In academia there has often been a focus on the fluidity of intent, though more recently a distinction with non-suicidal self-injury has been acknowledged.[23,24] The free text answers indicated that the inclusion of self-harm led to some confusion regarding intent, which may have influenced the answers provided. Clarity regarding suicidal intent would however have been likely increased the acceptability of “attempted suicide”.

Secondly, in recent years there have been a number of publications and media guidelines advising against use of words such as “commit”, “successful” or “failed” in combination with “suicide”, including by authors of this paper and Samaritans.[13–15,25,26] To our knowledge, this advice has been based on anecdotal evidence regarding the views of people affected by suicide. The survey was developed following debate about whether these views were representative, and the potential implications of advising against use of one particular term. It is possible that such previous publications have influenced the findings, particularly since some recruitment took place through social media channels associated with the authors and Samaritans. For example our supplementary findings indicate that “killed themselves” and “completed suicide” were considered acceptable by a greater proportion of respondents in the USA compared with Australia and UK, which may reflect USA guidelines advocating the use of these phrases. Overall, however, the study demonstrates mixed results for the term “commit suicide”.

Thirdly, whilst the baseline characteristics indicate participation from people with a range of backgrounds, the inclusion of a significantly greater proportion of younger females with higher levels of education suggests that the findings are more likely to represent this group. Furthermore, the findings may not be representative of all English-speaking countries, as responses from English-speaking countries other than Australia, the UK and the USA were limited. The supplementary analysis indicates that cultural differences may influence preferences, and therefore further research on this is warranted. However the key finding that “attempted suicide”, “took their own life”, “died by suicide” and “ended their life” were considered acceptable by most people holds true in the different countries.

Finally, when considering whether the nature of experiences affects perceived acceptability of terminology, it is pertinent to note that most participants were affected by suicide through more than one type of experience. These participants were not included in the sub-group analysis, which compared those who had *only* been affected by their own, someone else’s or professional experiences. There are also likely to be other differences in the nature of participants’ experiences within the three sub-groups, which we have not been able to ascertain but may influence their preferences.

### Comparison to other studies

The findings of this survey are in-keeping with an Australian Delphi study, which aimed to develop evidence-based guidelines for young people communicating about suicide via social media.[27] The Delphi study did not, however, require personal experience of suicide to join the professional and youth panels. The panels endorsed items that advised avoiding language that glorifies suicide, and avoiding terms with criminal connotations. Our findings indicate mixed views regarding whether the phrase “commit suicide” carries such connotations. However, both studies endorse use of “died by suicide”.

When making recommendations about language, a key consideration is the impact of language on behaviour. In the case of suicide, the impact on further suicidal behaviour and help-seeking is important and has been highlighted in our findings, with some raising concerns that “near miss” and “successful suicide” might encourage further suicidal behaviour by framing suicide as inevitable or as a positive outcome respectively. Participants in this study did not raise the possibility that language considered stigmatising might be protective against further attempts, as has previously been speculated.[20]

A recent randomised controlled trial examined framing effects of the use of different German-language descriptors: “suizid” (“suicide”), “freitod” (“free death”) and “selbstmord” (“self-murder”).[28] The analysis revealed that the term “freitod” increased support towards suicide amongst people suffering from incurable diseases. In contrast to some suggestions in our survey to include “chose to” or “decided to” within a descriptor, the authors of the German trial highlighted research countering the idea that suicide is a “free” and “rational” decision, and concluded that their findings supported use of the term “suizid”. The study also found that use of specific descriptors increased their use subsequently, a finding that was replicated in this study with a number of those selecting a descriptor stating that it was due to its familiarity, including the term “committed suicide”. Further research on the effect of English-language descriptors on attitudes and behaviour would be valuable. Additionally our survey highlights the need for investigation of language using a broader conceptual framework, which explores the inclusion of reference to the intent and suicide itself.

To conclude, variation in opinion exists amongst people affected by suicide regarding most phrases, often depending on contextual factors. Opinion about “commit suicide” was most divided. Both use of the phrase, and advice against use, were highly emotive for some participants. “Attempted suicide”, “took their own life”, “died by suicide” and “ended their life” were, however, considered acceptable by most participants, including those who considered “commit suicide” most appropriate. We would argue that academic and media guidelines should therefore promote the use of these phrases.

## Supporting information

S1 File Survey. (DOCX)

S2 File All responses (both complete and incomplete). (DOCX)

S3 File Responses by country: United Kingdom, United States of America and Australia. (DOCX)

S4 File Additional analysis relating to “committed suicide”. (DOCX)
