# Supplementary material for: Language use and suicide: An online cross-sectional survey
Source: PLoS One. 2019 Jun 13;14(6):e0217473. doi: 10.1371/journal.pone.0217473 (PMC6563960; doi:10.1371/journal.pone.0217473)
Supplement: S1 File — (DOCX) [file pone.0217473.s001.docx]

**S1 File**

**Survey**

**Page 1**

Participant information sheet

**Page 2**

1. What is your age?

18-29 years old

30-49 years old

50-64 years old

≥65 years old

1. How would you describe your gender?

Male

Female

Transgender male

Transgender female

Gender variant/non-conforming

Prefer not to say

Other (please specify)

1. What is your country of residence?

[Dropdown menu of countries provided by Surveymonkey]

1. What is the highest level of education that you have completed?

Primary Education

Secondary/Further Education (including GNVQ, BTEC, GCSE, A-level, IBDP, Le Bac, high school diploma)

Higher Education (including undergraduate degree, masters degree, PhD)

Other (please specify)

1. How have you been affected by suicide? (please tick all that apply)

A close friend or relative attempted or died by suicide

An acquaintance or colleague attempted or died by suicide

I have worked in a professional capacity (e.g. as a practitioner) with someone who has attempted or died by suicide

I have attempted suicide

I have experienced thoughts of suicide, but not acted on them

None – I have not had any experience of suicide either personally or others I know

Other e.g. research experience (please specify)

**Page 3/popup**

**Before you continue, we'd just like to check you're happy to carry on, and remind you of some organisations which can provide support if required:**

UK or Republic of Ireland

Samaritans

Phone: 116123

Email: jo@samaritans.org

Website: https://www.samaritans.org/

HOPELine UK

Phone 08000684141

Website: https://www.papyrus-uk.org/

CALM

Phone: 0800 58 58 58 (5pm-midnight daily)

Website: http://www.thecalmzone.net/

Survivors of Bereavement by Suicide

Phone: 0300 111 5065 (9am-9pm daily)

Website: https://uksobs.org/

For those outside the UK/ROI

Befrienders Worldwide
Website: [www.befrienders.org](http://www.befrienders.org)

Please [**click here**](https://www.iasp.info/resources/Crisis_Centres/) for a more comprehensive list of support organisations and helplines across the world.

**Please click 'Next' to continue with the survey.**

**Page 4**

**Please rate on a scale of 1 to 5 how acceptable you feel these phrases are to describe a situation where a person has performed an act of suicide or self-harm but has not died (1 – not acceptable; 5 – acceptable)**

**6. Cry for help**

**7. Suicidal gesture**

**8. Failed attempt at suicide**

**9. Attempted suicide**

**10. Unsuccessful suicide**

**11. Parasuicide**

**12. Near-miss**

**13. Non-fatal self-harm**

**14. Suicide survivor**

15. Please state which, if any, phrase from the list you feel is most appropriate to use [Drop down menu of options above]

16. Please explain your answer [free text field]

17. Please state which, if any, phrase from the list is least appropriate to use [Drop down menu of options above]

18. Please explain your answer [free text field]

**Page 5**

**Please rate on a scale of 1 to 5 how appropriate you feel these phrases are when describing a situation where a person has performed an act of suicide or self-harm and has died  (1 – not acceptable; 5 – acceptable)**

19. Died by suicide

20. Suicide victim

21. Successful suicide

22. Topped themselves

23. Ended their life

24. Completed suicide

25. Took their life

26. Committed suicide

27. Killed themselves

28. Fatal self-harm

**29.** Please state which, if any, phrase from the list you feel is most appropriate to use [dropdown menu of phrases above]

30. Please explain your answers [comment box]

31. Please state which, if any, phrase from the list is the least appropriate to use [dropdown menu]

**32. Please explain your answer** [comment box]

**Page 6**

33. Are there any other phrases that you think it would be more appropriate to use instead of those described in this survey?

**Page 7**

**There are no more questions. Thank you for completing the survey.**

If you have any questions about the survey please contact Dr Prianka Padmanathan [email address link] or Dr Dee Knipe [e-mail address link]. If you have any complaints please email research-governance@bris.ac.uk.

Please remember, if you feel you would like some help or support in relation to the issues raised in this survey, the following organisations are available to help:

UK or Republic of Ireland

Samaritans

Phone: 116123

Email: jo@samaritans.org

Website: https://www.samaritans.org/

HOPELine UK

Phone 08000684141

Website: https://www.papyrus-uk.org/

CALM

Phone: 0800 58 58 58 (5pm-midnight daily)

Website: http://www.thecalmzone.net/

Survivors of Bereavement by Suicide

Phone: 0300 111 5065 (9am-9pm daily)

Website: https://uksobs.org/

For those outside the UK/ROI

Befrienders Worldwide
Website: www.befrienders.org

Please [**click here**](https://www.iasp.info/resources/Crisis_Centres/) for a more comprehensive list of support organisations and helplines across the world.
