# Supplementary material for: Language use and suicide: An online cross-sectional survey
Source: PLoS One. 2019 Jun 13;14(6):e0217473. doi: 10.1371/journal.pone.0217473 (PMC6563960; doi:10.1371/journal.pone.0217473)
Supplement: S2 File — (DOCX) [file pone.0217473.s002.docx]

**S2 File**

**All responses (both complete and incomplete)**

Figure 1: Acceptability scores of terms describing non-fatal suicidal behaviour for all participants including those with incomplete responses (excludes outliers; ordered by medians, denoted by dashed line; 1=unacceptable 5=acceptable)

Figure 2: Acceptability scores of terms describing fatal suicidal behaviour for all participants including those with incomplete responses (excludes outliers; ordered by medians, denoted by dashed line; 1=unacceptable 5=acceptable)
