# Supplementary material for: Language use and suicide: An online cross-sectional survey
Source: PLoS One. 2019 Jun 13;14(6):e0217473. doi: 10.1371/journal.pone.0217473 (PMC6563960; doi:10.1371/journal.pone.0217473)
Supplement: S3 File — (DOCX) [file pone.0217473.s003.docx]

**S3 File**

**Responses by country:** **United Kingdom, United States of America and Australia**

**Non-fatal suicidal behaviour**

Figure 1: Acceptability scores of participants living in the United Kingdom (excludes outliers; medians denoted by dashed line; 1=unacceptable 5=acceptable)

Figure 2: Acceptability scores of participants living in the United States of America (excludes outliers; medians denoted by dashed line; 1=unacceptable 5=acceptable)

Figure 3: Acceptability scores of participants living in Australia (excludes outliers; medians denoted by dashed line; 1=unacceptable 5=acceptable)

**Fatal suicidal behaviour**

Figure 4: Acceptability scores of participants living in the United Kingdom (excludes outliers; medians denoted by dashed line; 1=unacceptable 5=acceptable)

Figure 5: Acceptability scores of participants living in the United States of America (excludes outliers; medians denoted by dashed line; 1=unacceptable 5=acceptable)

Figure 6: Acceptability scores of participants living in Australia (excludes outliers; medians denoted by dashed line; 1=unacceptable 5=acceptable)
