# Supplementary material for: Language use and suicide: An online cross-sectional survey
Source: PLoS One. 2019 Jun 13;14(6):e0217473. doi: 10.1371/journal.pone.0217473 (PMC6563960; doi:10.1371/journal.pone.0217473)
Supplement: S4 File — (DOCX) [file pone.0217473.s004.docx]

**S4 File**

**Additional analysis relating to “committed suicide”**

*Figure 3: Box plots of acceptability scores of those who selected “committed suicide” as their most or least preferred descriptor (excludes outliers; medians denoted by dashed line; 1=unacceptable 5=acceptable)*
